# Involvement of a Minimal Actin-Binding Region of *Spiroplasma citri* Phosphoglycerate Kinase in Spiroplasma Transmission by Its Leafhopper Vector

**DOI:** 10.1371/journal.pone.0017357

**Published:** 2011-02-22

**Authors:** Fabien Labroussaa, Marie-Pierre Dubrana, Nathalie Arricau-Bouvery, Laure Béven, Colette Saillard

**Affiliations:** INRA and Université Bordeaux UMR 1332 Biologie du fruit et pathologie, Villenave d'Ornon, France; Indian Institute of Science, India

## Abstract

**Background:**

*Spiroplasma citri* is a wall-less bacterium that colonizes phloem vessels of a large number of host plants. Leafhopper vectors transmit *S. citri* in a propagative and circulative manner, involving colonization and multiplication of bacteria in various insect organs. Previously we reported that phosphoglycerate kinase (PGK), the well-known glycolytic enzyme, bound to leafhopper actin and was unexpectedly implicated in the internalization process of *S. citri* into *Circulifer haematoceps* cells.

**Methodology/Principal Findings:**

In an attempt to identify the actin-interacting regions of PGK, several overlapping PGK truncations were generated. Binding assays, using the truncations as probes on insect protein blots, revealed that the actin-binding region of PGK was located on the truncated peptide designated PGK-FL5 containing amino acids 49–154. To investigate the role of PGK-FL5-actin interaction, competitive spiroplasma attachment and internalization assays, in which His_6_-tagged PGK-FL5 was added to Ciha-1 cells prior to infection with *S. citri,* were performed. No effect on the efficiency of attachment of *S. citri* to leafhopper cells was observed while internalization was drastically reduced. The *in vivo* effect of PGK-FL5 was confirmed by competitive experimental transmission assays as injection of PGK-FL5 into *S. citri* infected leafhoppers significantly affected spiroplasmal transmission.

**Conclusion:**

These results suggest that *S. citri* transmission by its insect vector is correlated to PGK ability to bind actin.

## Introduction

The plant pathogenic mollicute *Spiroplasma citri*, available in culture since 1971 [1], [2], has emerged as an outstanding model for studying spiroplasma interactions with its two experimental hosts: the periwinkle plant and the insect vector *Circulifer haematoceps* [3]. Due to its circulative and persistent transmission, *S. citri*, similarly to the other plant pathogenic mollicutes, has to complete a complex journey in the insect. Successful transmission of *S. citri* by the leafhopper depends mainly on the ability of spiroplasmas to pass through the insect gut cells, to multiply in various tissues, and finally to cross the salivary gland cells. The crossing of these different barriers doubtlessly requires protein interactions between the spiroplasma cells and cells of its insect host. The salivary gland invasion represents the essential and ultimate step. Thus the first cell-to-cell contact may be crucial for the efficient penetration of the salivary gland membrane. Following electron microscopy observations, it has been hypothesized that leafhopper transmission of spiroplasmas and more precisely the traversal of insect membranes was mediated by the recognition of specific membrane proteins, which led to a process of receptor mediated endocytosis [4], [5], [6]. Nevertheless, the mechanisms governing these crossings have not been conclusively determined. Earlier reports on *Spiroplasma kunkelii* describe tip structures piercing the basal laminae of midgut epithelial cells of the leafhopper vector *Dalbulus maidis* [6], [7]. In order to bind, penetrate and degrade the basal laminae, these tip structures or attachment organelles very likely contain specialized enzymes, receptors and/or adhesins as for human mycoplasmas [8], [9].

A few number of *S. citri* membrane or membrane-associated proteins potentially involved in leafhopper transmission, including the solute binding protein Sc76 [10], the P32 protein [11], [12], the adhesion relative proteins ScARPs [13] and spiralin [14] have been identified. Among these proteins, solely spiralin was described as interacting with host proteins. Indeed, spiralin acts as a lectin able to bind, *in vitro,* insect glycoproteins that may serve as receptors for the adherence of spiroplasmas [15].

Recently, we reported that confocal analysis focused on internalization and overall distribution of *S. citri* at the salivary gland level revealed spiroplasmas located along the actin microfilaments [16]. This co-localization was also observed with Ciha-1 cells, a leafhopper cell line from *Circulifer haematoceps*, experimentally infected by *S. citri* [17]. This preferential localization of spiroplasma cells suggested that invasion into host cells may involve interactions between *S. citri* proteins and the actin cytoskeleton. Among phytopathogenic mollicutes, the first interaction with actin was identified in ‘*Candidatus* Phytoplasma asteris’ (OY strain) and involved its immunodominant membrane protein (Amp). While this interaction has an unclear mechanism, it seems to be implicated in the insect-vector specificity [18]. The ability to bind to actin microfilaments is a characteristic feature that has been reported for many intracellular bacterial pathogens [19], [20] and might represent a general scheme for bacterial invasion into host cells.

For *S. citri,* we previously reported that phosphoglycerate kinase (PGK), a key enzyme in glycolysis which is thus classified as a cytosolic protein, unexpectedly bound actin and mediated the process leading to internalization of *S. citri* in eukaryotic cells [16]. Several reports indicate the presence of cytosolic proteins on surfaces of different bacteria but how these proteins are translocated to the surface, remains unanswered especially when conventional secretion or anchoring signals are absent. Thus PGK joins the group of enzymes including enolase [21], glyceraldehyde-3-phosphate dehydrogenase [22], and pyruvate dehydrogenase E1β subunit [23] that besides their apparent metabolic functions may have some other roles to play in bacteria.

PGK enzyme catalyzes the reversible transfer of a phosphate group from 1,3-bisphosphoglycerate to ADP, resulting in the formation of 3-phosphoglycerate and ATP. This function in the glycolysis requires the formation of a PGK ternary complex including its two ligands, 1,3-bisphosphoglycerate and ADP, bound to PGK on the N-terminal domain and C-terminal domain respectively. These two domains are connected by a conserved hinge region which allows the conformational change of the complex structure bringing the two ligands into close proximity during catalysis (Bernstein et al., 1997). Furthermore, the ADP/ATP molecule is well known to interact with actin and could thus play the role of a linking agent between these two proteins. The question whether the interaction between PGK and actin requires the ATP/ADP binding C-terminal moiety and/or the N-terminal 1,3-bisphosphoglycerate binding region remains opened. Aiming at elucidating this point, this study was carried out to identify the PGK parts that mediate binding to actin of *C. haematoceps* host cells and to determine the *in vivo* effect of the actin-binding region in the transmission process of spiroplasmas by its insect vector.

## Materials and Methods

### Bacterial strains, leafhoppers, and cell line culture

*Escherichia coli* strains DH10B and BL21 (DE3) were grown in Luria-Bertani (LB) medium and used to clone, express, and purify *S. citri* His_6_-tagged PGK and its truncated forms. *S. citri* GII3, originally isolated from its leafhopper vector *C. haematoceps* captured in Morocco [24], was cultivated in SP4 medium [25] at 32°C.

Healthy *C. haematoceps* were reared in an insect-proof cage on stock (*Matthiola incana*) plants at 30°C. Micro-injection of *S. citri* GII3 into *C. haematoceps* has been described previously [26].

The non-phagocyte cell line Ciha-1 from the *Cicadellidae C. haematoceps* was cultured at 32°C according to Duret *et al*. [17].

### Cloning, expression, and purification of PGK full length and PGK truncations

Expression and purification of *S. citri* His_6_-tagged PGK was previously described and PGK truncations were produced from the pET28 FL plasmid containing the *pgk* gene, in which the two UGA codons were replaced by TGG codons [16]. The individual truncated fragments *pgkFL1* to *pgkFL5*, were PCR amplified (Table 1) and the resultant DNA fragments were inserted in pET28a(+) vector (Novagen). The recombinant plasmids (pET28FL1 to pET28FL5) were used to transform *E. coli* DH10B. The desired sequence of the resulting plasmids was verified by sequencing the insert. Two µg of plasmid representing each truncation was used to transform *E. coli* BL21 (DE3). Transformants were selected on LB solid medium containing kanamycin (50 µg/ml) at 37°C.

**Table 1:** Primers used for constructions of His_6_-tagged PGK and PGK truncations^a^.

Protein	Primers	Sequence (5′ to 3′)^b^	Position^c^	Amino acid residue numero
PGK	PGK-FPGK-R	GTGAGAATTCGGATTTCATATGACAAAC TTATGAAGCTTTTATTTACTTTGAACAGC	−19 to +9	1–412
			1222–1250*	
PGK FL1	PGKpep-FPGKpep1-R	TGAGAAGGATTTGAATTCCATATGACAAAC CAAAACCTAAGCTTTTAGTTGTAACTTTTG	−18 to +9	1–302
			283–303	
PGK FL2	PGKpep-FPGKpep2-R	TGAGAAGGATTTGAATTCCATATGACAAAC CATTGTGATAAGCTTTTAAACATTTTTACTTC	−18 to +9	1–205
			590–615	
PGK FL3	PGKpep-FPGKpep3-R	TGAGAAGGATTTGAATTCCATATGACAAAC CCAATAAGCTTTTATGTTTTCCTTCAAAGG	−18 to +9	1–101
			883–906	
PGK FL4	PGKpep4-FPGK-R	GTAAAAATGAATTCCATATGGATTCTGCTTTAGG TTATGAAGCTTTTATTTACTTTGAACAGC	303-324	101-412
			1222–1250*	
PGK FL5	PGKpep5-FPGKpep5-R	TTGATAGAATTCCATATGGCACAAGAAGCAAAAG AATATTTCCCTAAAAGCTTTTAAGCAGAATC	147-168	49-154
			438–462	

a All regions were amplified using the respective mutagenic forward and reverse primers, as required.

b Introduced EcoRI, HindIII and NdeI sites underlined.

c upstream position of nucleotide from the adenine nucleotide of the starting ATG codon.

*11 nucleotides are downstream the stop codon localized at position 1239.

The expression of PGK truncations fused to the N-terminal hexahistidine sequence was carried out as previously described for entire PGK [16]. All proteins, purified by affinity chromatography using Ni^2+^-nitrilotriacetic acid (Ni-NTA) columns (Qiagen), were desalted using PD-10 columns according to manufacturer's instructions (GE Healthcare) and protein concentrations were estimated by the Bradford procedure. Two µg of each purified His_6_-tagged proteins were subjected to 5–15% linear gradient SDS-PAGE and visualized by gel staining with colloidal blue. Parallel gels were transferred onto membranes and blots were used for (i) immunoblotting performed with anti-His monoclonal (Mab) antibodies (1∶3000 dilutions, Sigma), (ii) far Western blotting carried out with a mixture of leafhopper cell proteins.

### Identification of PGK truncations that interact with leafhopper actin

Interactions between individual His_6_-tagged PGK truncations and leafhopper actin were detected by far Western experiments.

Equal amounts (2 µg) of His_6_-tagged PGK and its individual truncations were fractionated by a 5–15% linear gradient SDS-PAGE before transfer onto a nitrocellulose membrane for 45 min at 10 V according to the conditions described by Killiny *et al* [15]. Membrane blots were then overlaid with proteins prepared from Ciha-1 cells as follows: 5×10^6^ Ciha-1 cells, cultured into a 24 mm diameter well, were trypsinized with TrypLE (Invitrogen) and centrifuged at 5, 000 g for 5 min. Cells were transferred to a potter-Elvehjem-grinder with phosphate buffered saline (PBS) (2 mM KH_2_PO_4_, 8 mM Na_2_HPO_4_, 0.14 M NaCl, 2 mM KCl pH 7.4) containing 1 mM phenylmethanesulphonylfluoride (PMSF) and homogenized. Then the mixture was centrifuged twice for 1 min at 500× g. Protein concentration in the supernatant was determined by the Bradford procedure and aliquots of 500 µg of proteins were used as overlay for incubation with the blots of His_6_-tagged proteins. Interactions were revealed with rabbit polyclonal antibodies against actin (1∶600 dilution, Sigma) followed by goat anti-rabbit labelled with peroxidase (1∶50,000 dilution, Sigma). All the steps were conducted in the same conditions as those previously published [16].

All individual His_6_-tagged truncated proteins were also used as overlay on a leafhopper total protein blot. These far Western experiments were conducted as follows: 20 µg of total proteins prepared from Ciha-1 cell culture (equivalent to 4×10^4^ cells), prepared as described above were separated on 12,5% SDS-PAGE and transferred to nitrocellulose membrane. Then each blot was overlaid with 40 µg of individual PGK truncation and interaction with leafhopper actin was revealed using anti-His MAb (1∶3,000 dilution, Sigma) followed by peroxidase conjugate goat anti-mouse IgGs (1∶20,000 dilution, Sigma).

### Effect of recombinant PGK and its truncations on *S. citri* attachment to and entry into Ciha-1 cells

Monolayers of Ciha-1 cells grown in 24-well plates (2×10^5^ cells per well) were incubated with various concentrations of *S. citri* His_6_-tagged PGK and truncations PGK-FL4 and PGK-FL5 diluted in Ciha-1 cell culture medium [17]. Cells without PGK treatment represent positive control. Cells treated with 400 µg/ml of BSA were also included as a control. Each assay was carried out in triplicate. After incubation with proteins for 2 h at 32°C, the cells were infected with a 100-µl *S. citri* suspension at a multiplicity of infection (MOI) of 15 to 30. After infection with *S. citri*, spiroplasma attachment to and internalization into Ciha-1 cells were estimated by counting colonies on solid SP4 medium as described previously [16]. Briefly, for attachment assay, cells were incubated for 4 h at 4°C allowing spiroplasma adhesion but at this temperature eukaryotic cell processes required for internalization were inhibited. Then cells were washed three times with 500 µl of Schneider's Drosophila medium to remove any spiroplasmas that had not attached to the monolayer. After trypsinization for 10 min at 32°C with TrypLE^TM^ (Invitrogen), dilutions of the cells associated with adherent spiroplasmas were directly plated onto solid SP4 medium. After incubation at 32°C for 1 week, the number of spiroplasma colonies on each plate was counted to estimate the number of Ciha-1 cells that had adherent spiroplasmas. Relative percentage of adhesion was calculated as follows: [(number of CFU for cells with protein treatment/number of CFU for untreated control cells) ×100%].

The invasion assay was conducted similarly than the attachment test except that, after infection with a 100-µl *S. citri* culture for 18 h at 32°C, a gentamicin protection assay was used. Briefly, cells were incubated with 1 ml of fresh culture medium containing 400 µg/ml of gentamicin (10 fold the minimum inhibitory concentration) during 3 h at 32°C in order to kill extracellular bacteria. The cells were thereafter washed three times with 500 µl of Schneider's Drosophila medium followed by three additional washes with PBS. After washes, a 100-µl aliquot of the last wash was plated onto SP4 medium to ensure that all extracellular bacteria had been killed.

### *S. citri* experimental transmission assays

Female leafhoppers were microinjected with 0.1 µl of a spiroplasma culture (10^8^ spiroplasmas/ml) and caged on healthy stock (*Matthiola incana)* plants at 30±2°C as previously described [26]. At day 4 after injection when the highest titer of spiroplasma cells in the insect hemolymph was reached (10^6^ spiroplasma cells/insect), they were microinjected again with 0.2 µg of His_6_-tagged PGK or truncations (PGK-FL4 or PGK-FL5) in phosphate buffer (8 mM NaH_2_PO_4_, 2 mM Na_2_HPO_4_ [pH 7,4]). Injections with phosphate buffer and with a His_6_-tagged protein (subunit of *Mycoplasma mycoïdes* SC ATPase) were included as controls in the experiment. Then, for each tagged protein or control, injected insects were randomly divided in subgroups of 3 among Eppendorf® tubes on which a Parafilm® membrane separated the leafhoppers from SP4 medium as previously described [26]. When feeding through the Parafilm® membrane, infected leafhoppers injected *S. citri* into the medium. After 24 h at room temperature, the SP4 medium was collected and incubated at 32°C. After one week, the yellow colour in the tube, indicating the growth of *S. citri,* was noticed and the presence of spiroplasmas was verified with optical microscopic observations.

### Statistical analyses

For attachment to and invasion of *S. citri* into Ciha-1 cells, Student's t test was used. For *S. citri* transmission assay, a chi-square test (two-tailed) was performed to identify significant differences.

## Results

### Production of recombinant PGK truncations

**Figure 1:**
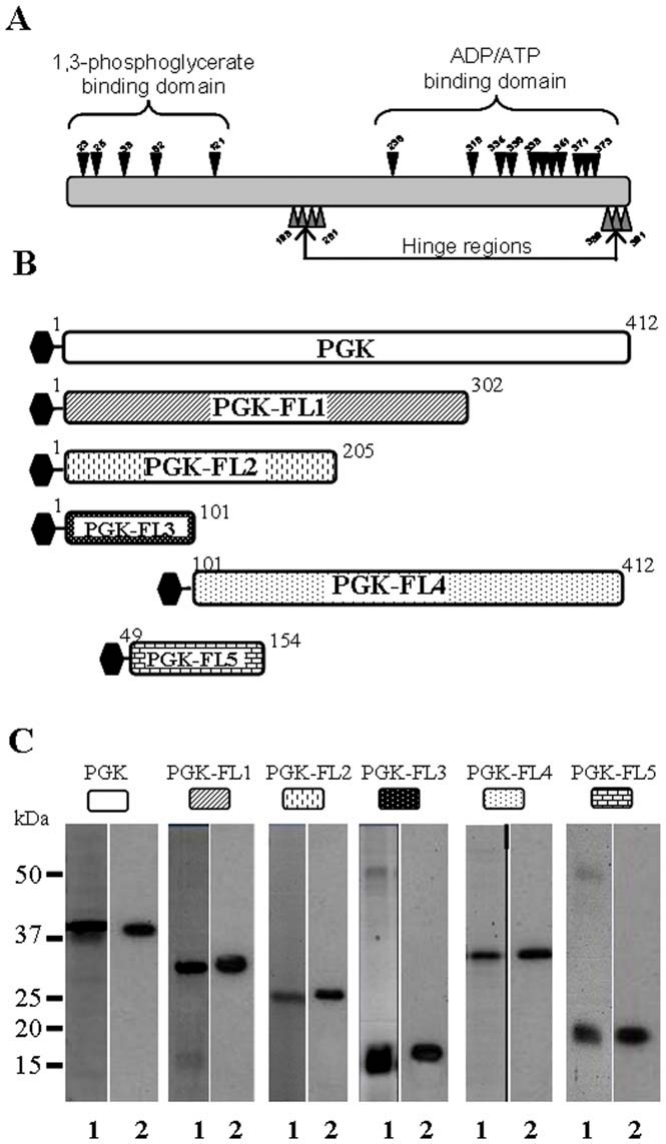
Construction and purification of the truncated PGK proteins. (A) Schematic illustration of PGK protein showing positions of amino acids interacting with its two substrates in glycolysis. The N-terminal part of the PGK and more precisely, amino acids D23, N25, R38, H62 and R121 are implicated in 1,3-biphosphoglycerate binding. The C-terminal part of the PGK containing amino acids G236, G310, N334, P336, G338 to E341 and G371 to T373 are implicated in ADP binding. Hinge regions containing amino acids L198 to P201 and V389 to T391 connect the two others domains of PGK and are responsible for inducing conformational change during catalysis. (B) Schematic illustration of the PGK and deletion constructs. Full-length PGK protein (amino acids 1 to 412) was deleted from C-terminus and PGK-FL1 (amino acids 1 to 302), PGK-FL2 (amino acids 1 to 205), PGK-FL3 (amino acids 1 to 101), were produced. N-terminal deletion produced PGK-FL4 (amino acids 101 to 412). PGK-FL5 (amino acids 49 to 154) was constructed by deleting both amino terminal and carboxy-terminal ends of PGK. (C) All His_6_-tagged proteins were purified as described in Materials and Methods, and subjected to SDS-PAGE (lane 1). Parallel gels were transferred onto nitrocellulose membranes. Saturated blots were probed with anti-His monoclonal antibodies (lane 2).

The predicted amino acids on *S. citri* PGK involved in substrate binding domains were identified by searches against the conserved domain databases [27] and are indicated on figure 1A.

In an attempt to identify the actin-binding region of PGK, we generated by deletion of the carboxyl-terminal region of the PGK (PGK full length: 412 amino-acids) three truncated polypeptides of varying lengths namely PGK-FL1 (302 aa), PGK-FL2 (205 aa) and PGK-FL3 (101 aa) (Figure 1B). The latter fragment corresponded to the shortest fragment bearing the 1,3-bisphosphoglycerate binding domain that was tested (Figure 1A). PGK-FL4 (312 aa) was the result of the deletion of the first hundred PGK amino acids and represented the PGK ATP/ADP binding site-containing, C-terminal part. A truncated form of PGK-FL2 containing amino acids 49-154 designed PGK-FL5 (106 aa) was also produced (Figure 1B). His_6_-tagged PGK and all the His_6_-tagged truncated proteins were expressed in *E. coli* and purified. His_6_-tagged proteins, PGK, PGK-FL1, PGK-FL2, PGK-FL3, PGK-FL4 and PGK-FL5 have respectively predicted molecular masses of 44.0, 33.7, 23.5, 12.2, 34.0, 12.5 kDa. On the colloidal blue-stained polyacrylamide gel, His_6_-tagged PGK, and His_6_-tagged truncations PGK-FL1, PGK-FL2 and PGK-FL4 matched their predicted molecular masses (Figure 1C, lanes 1). His_6_-tagged PGK-FL3 and PGK-FL5 migrated higher than their predicted molecular masses of 12.2 and 12.5 kDa and preparations of these two truncations were slightly contaminated by other *E. coli* proteins (Figure 1C, lanes 1). However, anti-His Mab recognized only the tagged proteins in all cases in spite of protein contaminations in PGK-FL3 and PGK-FL5 preparations (Figure 1C, lanes 2).

### Identification of the minimal PGK actin-binding region

**Figure 2:**
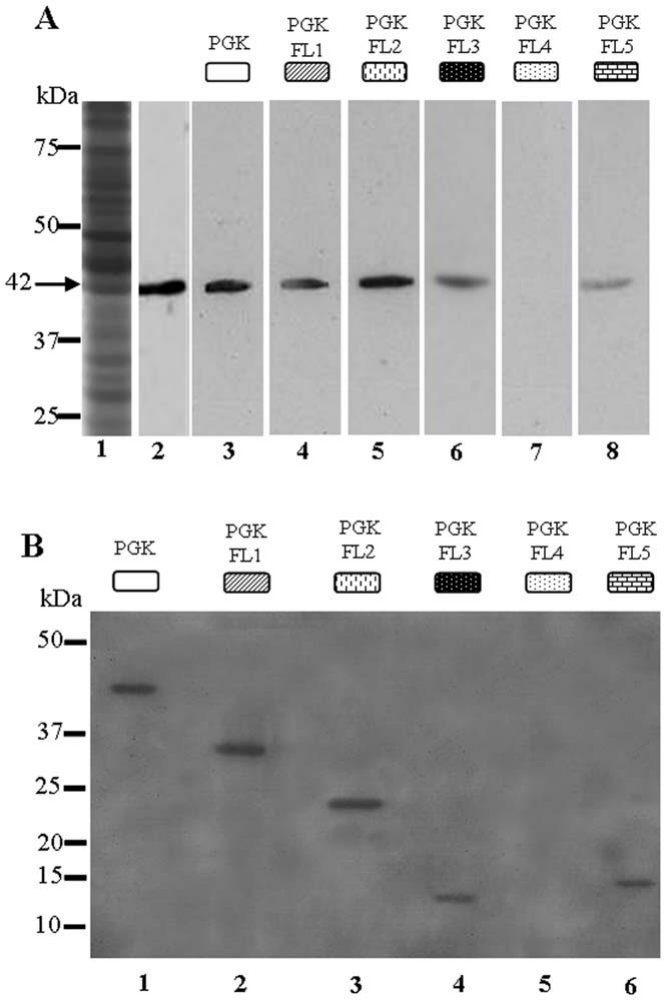
Determination of the minimal PGK region required for actin binding activity. (A) Far Western experiment performed on Ciha-1 cell proteins overlaid with His_6_-tagged PGK and His_6_-tagged PGK truncations. Lane 1, gel electrophoresis pattern of Ciha-1 cell proteins stained with colloidal blue. Lane 2, proteins from Ciha-1 cells were probed with rabbit polyclonal antibodies against chicken actin followed by immunological detection. The band at 42 kDa reflects the presence of actin in the protein mixture. Lanes 3 to 8, blots from Ciha-1 cell proteins were overlaid with PGK or individual PGK truncations. His_6_-tagged PGK and His_6_-tagged PGK-FL1 to PGK-FL3 and PGK-FL5 were bound to actin (lanes 3, 4, 5, 6, 8). With His_6_-tagged PGK-FL4, no binding signal was observed (lane 7). (B) Far-western experiments performed on PGK and its truncations overlaid with Ciha-1 cell proteins. Blots of His_6_-tagged PGK and His_6_-tagged PGK truncations were probed with the mixture of Ciha-1 cell proteins containing actin. Binding was detected with rabbit anti-actin antibodies followed by goat anti-rabbit antibodies labelled with peroxydase. Binding signals were observed with PGK and PGK-FL1 to PGK-FL3 and PGK-FL5 (lanes 1, 2, 3, 4, 6). No binding was observed with PGK-FL4 (lane 5).

To identify the truncated proteins that displayed a significant affinity for actin, a far Western assay was performed on a blot of Ciha-1 cellular proteins overlaid with individual truncated PGK proteins. Ciha-1 cell protein mixture was visualized on a 12.5% colloidal blue-stained polyacrylamide gel (Figure 2A, lane 1), and the presence of actin in the Ciha-1 cell protein mixture was confirmed by Western blotting using anti-actin antibodies (Figure 2A, lane 2). As shown on Figure 2A (lanes 3, 4, 5, 6, 8) for PGK, PGK-FL1 to FL3 and PGK-FL5 truncations, significant binding signals located at approximately 42 KDa corresponding to actin molecular mass were revealed by anti-His MAb. No signal was observed with PGK-FL4 (Figure 2A lane 7). From these results we deduced that the region in PGK from amino acid 49 to 101 possessed the capacity to bind actin.

To verify these findings, a second far Western assay was performed in which a blot of His_6_-tagged PGK or its truncations was overlaid with a Ciha-1 cells protein mixture (Figure 2B). Binding signals between PGK, its truncations and leafhopper actin were revealed by polyclonal antibodies against actin. As expected, an interaction signal was detected between PGK and actin (Figure 2B, lane 1). Truncation of PGK from the N-terminal (1–101), resulting in PGK-FL4 truncated protein (101 to 412), abrogated the binding activity (Figure 2B, lane 5), suggesting that this region played an important role for PGK interaction with actin. Binding signals detected with PGK-FL1, PGK-FL2 and PGK-FL3 (Figure 2A, lane 2, 3, 4) indicated that truncation from the PGK carboxyl-terminal had no effect on the actin-binding capacity of the PGK moieties. Thus, the 101-amino acid N-terminal region of PGK-FL3 was required for actin binding. The signal observed with PGK-FL5 (amino acid 49 to 154) (Figure 2B, lane 6) confirmed that an actin-binding site was located on this truncation. From these results, we conclude that the region from amino acids 49 to 101 is the minimal region that possessed the capacity to bind actin.

### Competitive binding assay with *S. citri* and truncated PGK proteins

**Figure 3:**
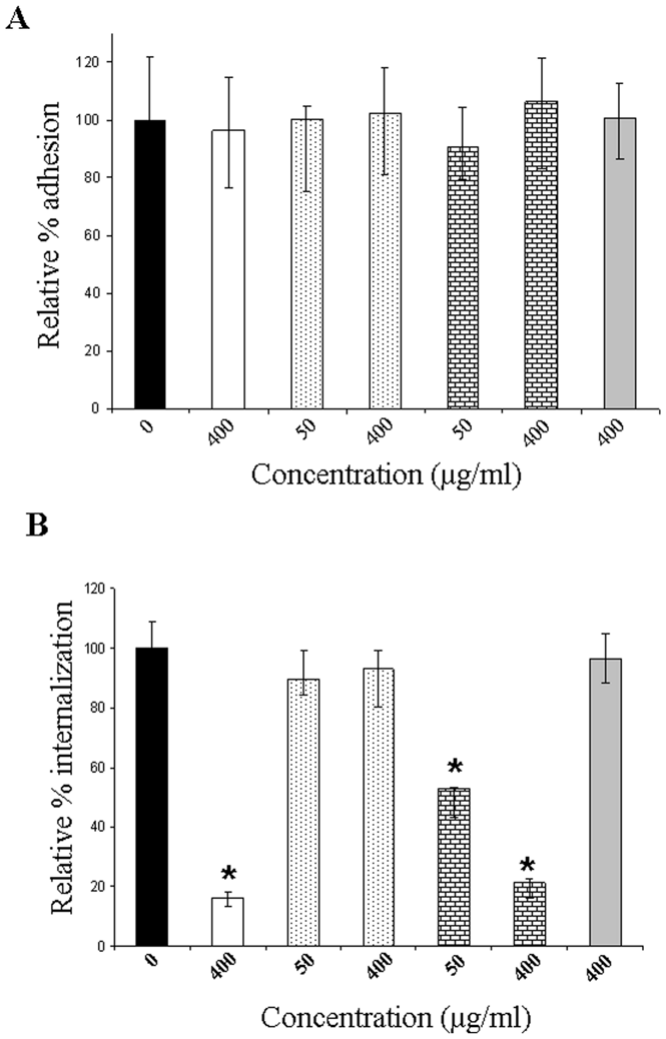
Competitive assays between recombinant PGK or its truncations and *S. citri* for attachment to and entry into Ciha-1 cells. Monolayers of Ciha-1 cells were incubated 2 h at 32°C with 400 µg of His_6_-tagged PGK (white bars), 50 and 400 µg of His_6_-tagged PGK-FL4 (spot-filled bars), 50 and 400 µg of His_6_-tagged PGK-FL5 (brick-filled bars), 400 µg of BSA (gray bars). Untreated cells infected in the same conditions were the positive controls (black bars). Each value represents the mean of two independent triplicate assays. Vertical lines represent standard error of the mean. (A) Effect of PGK treatment on *S. citri* attachment to Ciha-1 cells. After infection with *S. citri* for 4 h at 4°C, the cells were washed, trypsinized and plated on SP4 solid medium. After spiroplasma growth at 32°C, the number of colonies was counted to evaluate the number of cells associated with adherent spiroplasmas. Student's t test was carried out and no statistical differences were found. (B) Inhibition of *S. citri* internalization into Ciha-1 cells by a PGK treatment. Following infection with *S. citri* for 18 h at 32°C, the cells were treated with gentamicin (400 µg/ml) during 3 h at 32°C for killing attached spiroplasmas. Then the cells were trypsinized, plated on SP4 medium, for counting the infected cells. Significant differences between *S. citri* internalization following His_6_-tagged PGK, or PGK-FL5 treatment, and positive control without any protein treatment (*P*<0.001, Student's t test) are indicated by asterisks.

To determine whether the minimal actin-binding region of PGK (PGK-FL5) is sufficient to interfere with the interactions between spiroplasma PGK and host actin, thus preventing spiroplasma from entering into insect cells, competitive adhesion and invasion assays with PGK truncations were performed. No difference was observed between the relative percentage of adhesion obtained in Ciha-1 cells without any treatment or treated with BSA prior to infection with *S. citri* (Figure 3A). Competition of His_6_-tagged PGK (400 µg/ml) with Ciha-1 cells before *S. citri* infection did not reduce the binding of *S. citri* to cells. Competition with 50 and 400 µg/ml of either PGK-FL4, which has no actin-binding activity, or PGK-FL5, which is the minimal actin-binding region, did not noticeably alter the spiroplasmas attachment to cells.

Spiroplasma invasion of Ciha-1 cells was estimated similarly to the attachment test after a gentamicin protection assay. As shown in Figure 3B, the percentage of internalization of *S. citri* in cells incubated with BSA (400 µg/ml) was close to those of the positive controls. Competition with His_6_-tagged PGK (400 µg/ml) showed the greatest inhibition rate in invasion (85%). Preincubation with two concentrations of PGK-FL4 did not significantly reduce the invasion of *S. citri* in Ciha-1 cells. In contrast, preincubation with 50 and 400 µg/ml of PGK-FL5 significantly reduced *S. citri* internalization into Ciha-1 cells by 45% and 80% respectively similarly to preincubation with the entire His_6_-tagged PGK.

### Effect of PGK or PGK truncations on *S. citri* transmission

To further assess the effect of PGK in *S. citri* transmission by leafhoppers we compared the ability of *S. citri* to be inoculated in SP4 medium by infected leafhoppers injected with phosphate buffer versus infected leafhoppers injected with PGK or PGK truncations. Two hundred and fifty seven groups of 3 infected leafhoppers injected with phosphate buffer (positive controls) were allowed to feed for 24 h on SP4 medium. As shown on Table 2, a spiroplasma culture was obtained for 149 out of the 257 batches. Thus, in 58% of the batches, insects succeeded in inoculating *S. citri* into SP4 medium. A relative percentage of 100 was attributed to this maximal transmission rate. There was no significant difference in the percentage of *S. citri* inoculation in SP4 medium between the controls and infected insects microinjected with a His_6_-tagged protein or PGK-FL4 (96.5% and 100%). Injection with the His_6_-tagged PGK protein or PGK-FL5 peptide in the *S. citri* infected leafhoppers resulted in a significant reduction of bacterial inoculation in SP4 medium, compared with controls. The percentage of leafhoppers which introduced *S. citri* in SP4 medium was not significantly different following injection with the His_6_-tagged PGK protein or the PGK-FL5 peptide (51.7% and 58.6%, respectively).

**Table 2:** Effect of PGK and its truncations PGK-FL4 and PGK-FL5 on *S. citri* transmission.

	Number of independant experiments	Number of batches (3 leafhoppers per batch)	Number of batches with positive culture	Percentage of batches with positive culture	Relative percentage of batches with positive culture
Phosphate buffer	12	257	149	58	100
His_6_ control	3	100	56	56	96.5
PGK	8	176	52	30*	51.7*
PGK FL4	3	82	48	58	100.9
PGK FL5	4	112	38	34*	58.6*

*p<0.0001, chi-square test (two-tailed).

## Discussion

In this study we identified regions of *S. citri* PGK protein that interact with leafhopper actin and attempted to determine whether PGK, through its binding with actin, play a role in transmission of *S. citri* by the leafhopper *C. haematoceps*.

Based on our experimental data using PGK truncated derivatives, we determined that the region of the PGK which binds to actin was located on the PGK-FL3 truncation, corresponding to the N-terminal part of PGK (amino acids 1 to 101). Four out of the 5 well defined substrate binding sites of PGK (D22, N24, R37, H60) mediating interaction with phosphoglycerate during glycolysis are present on this fragment [27], [28]. In contrast, the PGK-FL4 fragment (amino acids 101 to 412) corresponding to the C-terminal part of PGK, exhibited no binding to actin. This PGK-FL4 fragment mainly includes the ADP/ATP binding sites and the catalytic site. The fact that the C-terminal part of PGK is not required for actin-binding capacity of PGK seems to prove that the interaction between PGK and actin is not mediated by ATP/ADP. To determine more precisely the actin-binding region, we constructed a PGK-FL5 truncation with an overlapping region of 53 amino acids with PGK-FL4 and in which the first 48 amino acids of PGK-FL3 were missing.

Interestingly, PGK-FL5 repeatedly showed a marked decrease in its actin-binding ability (Figure 2A lane 8), especially compared to PGK-FL1, PGK-FL2, and PGK-FL3 (Figure 2A lanes 4, 5, 6). As no significant difference in band intensity was visualized on the reciprocal far-Western (figure 2B lanes 4 and 6) the difference in signal levels between PGK moieties could depend on the relative concentrations of actin and PGK truncations. A difference in actin-binding efficiency of PGK-FL5 and PGK-FL3 could be explained by the occurrence of two distinct actin-interacting sites located in PGK N-terminal part, the first one being specific to PGK-FL3 and located within the amino acids 1-48, and the second site being common to both fragments within amino acids 49-101. Based on sequence similarity, the molecular model proposed for *S. cerevisiae* PGK [29] was used to predict the surface accessibility of the two hypothetical actin-binding domains in *S. citri* PGK. In the modelled *S. cerevisiae* PGK N-terminal part, two blocks of amino acids (M27 to E52 and K63 to Q88) were found to be localized side by side with a majority of the amino acids residues located at the outer surface of the molecule (data not shown). In *S. citri* PGK, the surface exposure of the corresponding amino acids residues would allow their interaction with actin. In addition, except for R37, amino acids that are believed to participate in actin-binding differ from those involved in PGK interactions with its substrate 1,3-biphosphoglycerate.

Previously we have described that PGKs from *S. citri* and *S. cerevisiae* do not interfere with adhesion of spiroplasmas to Ciha-1 cells but inhibit the internalization in the cells in a dose dependant manner [16]. In this study, as expected, PGK-FL4 and PGK-FL5 truncations or PGK full length had no effect on spiroplasmas adhesion to Ciha-1 cells. The fact that PGK-FL5, the minimal actin-binding region, conserved the inhibitory activity on the internalization process reinforces the hypothesis that the actin-binding efficiency of PGK is directly related to the relevance of such an interaction in the internalization process. This is also confirmed by the fact that PGK-FL4, which has no effect on internalization, does not possess any actin-binding activity.

These results prompted us to speculate that PGK and PGK-FL5 fragment are candidates for playing a role in transmission of *S. citri*. The penultimate event before the infectious bite in the phloem is invasion of *C. haematoceps* salivary glands by the spiroplasmas. To ascertain the role of PGK and PGK-FL5 truncation peptide in salivary gland invasion we developed an *in vivo* spiroplasma invasion blocking assay. Infected leafhoppers were injected with phosphate buffer or tagged proteins on the 4th day after infection. This time period corresponds to the maximum of spiroplasmas in insect [10] and a large majority of them would be expected to still be circulating in the haemolymph that is the main place of *S. citri* multiplication. Because transmission rates of *S. citri* between infected leafhoppers treated with His_6_-tagged PGK-FL4 and those of control groups (phosphate buffer or tagged protein) were similar, it is highly unlikely that PGK-FL4 inhibits the ability of spiroplasmas to enter in salivary glands. The infected leafhoppers injected with PGK or with PGK-FL5 displayed an average reduction of almost 50% in the transmission of *S. citri* when compared with controls. Therefore PGK-FL5 truncation, with its minimal actin-binding region, sufficient to block the entry of spiroplasmas into Ciha-1 cells, plays an important role in transmission. The competition between exogenous PGK and PGK-FL5 with the spiroplasmas could reduce the available number of target sites that participate in entry of spiroplasmas in salivary glands.

In summary, our results suggested a correlation between the PGK-actin interaction and the inhibitory effect during internalization in insect cells. In agreement with these data, as we show with our *in vivo* experimental model, *S. citri* could use this interaction for crossing salivary glands barrier and completing its life cycle in insects.
